# Clinical Value of Circulating Angiopoietin-like Protein 8/Betatrophin Levels in Patients with Acute Pancreatitis

**DOI:** 10.3390/medicina61040708

**Published:** 2025-04-11

**Authors:** Perihan Ozkan Gumuskaya, Emine Yildirim, Ozgur Altun, Hafize Uzun

**Affiliations:** 1 Department of Internal Medicine, Prof. Dr. Cemil Taşcıoğlu City Hospital, University of Health Sciences, 340110 Istanbul, Turkey; ozgurakademik@gmail.com; 2 Department of General Surgery, Faculty of Medicine, Istanbul Atlas University, 34403 Istanbul, Turkey; opdreyildirim@gmail.com; 3 Department of Medical Biochemistry, Faculty of Medicine, Istanbul Atlas University, 34403 Istanbul, Turkey; huzun59@hotmail.com

**Keywords:** acute pancreatitis, angiopoietin-like protein 8, betatrophin, inflammatory mediators

## Abstract

*Background and Objectives*: Acute pancreatitis (AP) is an inflammatory disorder of the pancreas, with severe cases linked to a higher mortality rate. The prognosis of AP is influenced by factors such as necrosis, secondary infections, and organ failure. Tissue damage in AP is driven by the activation of leukocytes and the release of inflammatory mediators. Angiopoietin-like protein 8 (ANGPTL8), also known as betatrophin, is a recently discovered protein that regulates lipid metabolism. This study aimed to investigate the relationship between ANGPTL8 levels and disease severity in AP patients, and to explore the potential of ANGPTL8 as a biomarker. *Materials and Methods*: This prospective study included 50 patients diagnosed with AP who were admitted to the Department of Internal Medicine at Dr. Cemil Taşcıoğlu City Hospital between September 2021 and February 2022. Additionally, 39 healthy volunteers who underwent a check-up at the same hospital served as the control group. The Glasgow–Imrie (GI) score was used to assess the severity of pancreatitis. *Results*: ANGPTL8 levels were found to be significantly lower in the AP group compared to the control group, with a statistically significant correlation between ANGPTL8 levels and the severity of AP (*p* < 0.05). The cut-off level of ANGPTL8 based on the GI score was determined to be 70.9 ng/L. The GI score for ANGPTL8 was 0.749 (95% CI: 0.606–0.861) (*p* < 0.001). The overall cut-off value for ANGPTL8 was 179.2 ng/L, with an overall classification rate of 0.936 (95% CI: 0.864–0.977) (*p* < 0.001). *Conclusions*: This study demonstrates that ANGPTL8 levels vary between patients with and without AP, with lower levels observed in AP patients. Our research is the first to identify decreased ANGPTL8 levels as an independent predictor of AP severity. ANGPTL8 may play a crucial role in regulating inflammation or metabolic dysfunction in AP. However, further studies are needed to confirm these findings in larger populations and investigate ANGPTL8’s mechanistic role in AP. Longitudinal studies could help determine whether ANGPTL8 levels act as a biomarker for disease progression or treatment response, potentially paving the way for targeted therapies to improve outcomes for AP patients.

## 1. Introduction

Acute pancreatitis (AP) is an inflammatory disease with high mortality and morbidity and characterized by self-digestion of the pancreas due to activation of pancreatic enzymes in the pancreatic gland for various reasons. It is accompanied by typical abdominal pain with high serum amylase and lipase enzyme levels [1]. The exact mechanisms of AP are not entirely known; activated leukocytes generated in AP and the release of inflammatory mediators contribute to tissue damage. The Ranson, Revised Atlanta criteria, Acute Physiology and Chronic Health Evaluation (APACHE) II score, Multiple Organ System Score (MOSS), and Modified Glasgow score systems are used to determine the clinical severity and prognosis of AP. According to the Revised Atlanta criteria [2], AP is histopathologically classified into interstitial, edematous, and acute necrotizing types and into mild, moderate, and severe categories according to disease severity. Mild AP is without local or systemic complications or organ failure. Moderate AP involves transient organ failure resolving within 48 h and/or local–systemic complications that last less than 48 h. Severe AP is characterized by persistent organ failure affecting one or more organs [2].

Angiopoietin-like peptide 8 (ANGPTL8) is also called betatrophin [3]. This protein is synthesized in hepatic and adipose tissues. It is a member of the angiopoietin-like protein family and involved in glucose metabolism, lipid metabolism and energy homeostasis. It is believed to be associated with diabetes mellitus (DM) [4]. ANGPTL8 is an important cytokine, which is significantly increased in type 2 DM (T2DM), obesity, and metabolic syndrome (MetS) [5]. ANGPTL8 is a newly discovered protein which is involved in the regulation of lipid metabolism [6] and lipoprotein lipase (LPL) regulation; LPL converts triglycerides into fatty acids, and these are taken into tissues like the muscles of the skeleton [7]. ANGPTL8 inhibits the LPL, which clears triglycerides from blood. This adipokine also promotes pancreatic beta cell proliferation and expands beta cell mass [8,9]. In humans, its main expression site is the liver, but subsequent studies have also found relative expression in adipose tissue [10,11].

Leukotriene B4 (LTB4), lipoxin A4 (LXA4), and resolvin D1 (RvD1) have been used in the etiologic investigation of AP due to gallstones [12], and RvD1 has also been reported to be a potential serum biomarker to differentiate between pancreatic ductal adenocarcinoma and AP [13]. The promising role of IL-6 as a prognostic marker suggests that it could be added as a routine marker on admission in children with AP [14]; controlled nutrition status (CONUT) and prognostic nutrition index (PNI) scores have shown promise in predicting necrotizing pancreatitis in patients presenting to the emergency department with AP and may serve as prognostic indicators for mortality in AP patients [15].

Understanding the role of ANGPTL8 in AP could provide insights into the underlying pathophysiology and its potential as a predictive marker for disease severity. If ANGPTL8 is shown to be a reliable indicator of disease progression, it could help in the design of new diagnostic tools, allowing for earlier detection of severe AP, more accurate prognosis, and personalized therapeutic interventions based on individual risk profiles. Additionally, understanding its mechanistic role in regulating inflammation and metabolism may open doors for targeted therapies to prevent or alleviate severe complications associated with AP. The aim of this study was to examine the association of ANGPTL8 levels with disease severity in patients with AP and to investigate whether ANGPTL8 can be used as a potential biomarker. Furthermore, how ANGPTL8 may modulate the inflammatory and metabolic processes of AP and how this mechanism may affect the course of the disease are also important focal points of the study. These findings may help to develop new diagnostic tools and better classify patients with respect to severe AP.

## 2. Materials and Methods

### 2.1. Ethical Approval

All procedures performed in the study involving human participants were in accordance with the ethical standards of the institutional and/or national research committee and with the 1964 Helsinki declaration and its later amendments or comparable ethical standards. Approval for this study was granted by the Prof. Dr. Cemil Taşcıoğlu City Hospital Ethics Committee for Clinical Studies in August 2021 (Approval date: 2 August 2021/257; Number: 48670771-514.10).

### 2.2. Data Collection

The research was designed as a prospective study and included 50 patients hospitalized with a diagnosis of AP in Prof. Dr. Cemil Taşcıoğlu City Hospital between September 2021 and February 2022. In the control group, who were age- and gender-matched with the patients, 39 individuals who were admitted to the check-up center of our hospital for routine controls and who did not have chronic diseases or active infections were included. Demographic characteristics and biochemical parameters of patients hospitalized for AP were obtained from the hospital’s electronic database system. All these patients met the study criteria. These patients underwent regular monitoring to assess their progress, detect possible complications, and address any changes in their health, as ongoing follow-up is vital for their condition. This continuous care is essential for effective management and to prevent further deterioration.

The study inclusion criteria were as follows: (i) having given consent to participate in the study, (ii) not having any chronic diseases. (iii) being over 18 years old and less than 70 years old, (iv) not having any drug usage or alcohol consumption, (v) not having hypertriglyceridemia, diabetes mellitus, or metabolic syndrome.

The study exclusion criteria were defined as follows: (i) refusal to participate in the study, (ii) patients with chronic diseases, (iii) patients with hypertriglyceridemia, (iv) patients with obesity. The patients underwent clinical and biochemical laboratory evaluations.

Data were obtained from each patient with a diagnosis of AP in respect of medical history, age and gender, body mass index (BMI), and laboratory data including amylase, lipase, C-reactive protein (CRP), albumin, calcium, international normalized ratio (INR), complete blood count, creatinine, alanine transaminase (ALT), direct bilirubin (D.Bil.), triglyceride (TG), and glucose. BMI is defined as the body mass divided by the square of the body height and is expressed in units of kg/m^2^. The pancreas was assessed by computed tomography. Patients who had AP due to hypertriglyceridemia or alcohol consumption were not included in the study. All patients were questioned about previous medical diagnosis of any other chronic disease and if there were any, removed from the study.

AP was diagnosed and classified based on the 2012 Revised Atlanta Classification [2]. Patients meeting at least two of the diagnostic criteria—abdominal pain consistent with a typical pancreatitis attack, serum amylase and lipase levels exceeding three times the upper limit of normal, and imaging findings indicative of pancreatitis on ultrasound or computed tomography (CT) or magnetic resonance imaging (MRI)—were considered to have AP. The AP group consisted of patients who presented to our clinic with abdominal pain. In addition to the Atlanta Classification, the Glasgow–Imrie (GI) score was used to evaluate the severity of pancreatitis [16]. It was calculated using eight laboratory parameters including pO2, age, neutrophil count, calcium, blood urea nitrogen, lactate dehydrogenase, aspartate aminotransferase (AST), albumin, and glucose.

GI scoring was calculated according to the following scoring system, in which each parameter received 1 point if it was compatible with the given value, and if the total score was ≥3, AP was considered severe, and if <3, it was considered not severe. AP patients were considered as severe and non-severe according to the GI score, not mild, moderate, or severe.
pO2 < 60 mmHg1 pointAge > 55 years1 pointNeutrophil count > 15,000 µ/L1 pointCalcium < 8 mg/dL1 pointUrea > 16 mmol/L1 pointLDH > 600 u/L1 pointAST > 200 u/L1 pointAlbumin < 32 g/L1 pointGlucose > 180 mg/dL1 pointModified Glasgow–Imrie severity criteria for AP.

### 2.3. Collection of Samples

Venous blood samples were taken from the subjects for analysis 6 h after the onset of pain (this is the onset of AP). Venous blood samples obtained from the participants were centrifuged at 4000 rpm for 5 min, and all biochemical parameters were analyzed in serum samples on the same day. Serum samples for serum ANGPTL8 measurement were stored at −80 °C until the study.

### 2.4. Measurement of Serum ANGPTL8 Levels

Serum ANGPTL8 levels were assessed using commercial ELISA assays (human BT-Lab kits) and a NUVE EN 500 incubator (Robonik, Mumbai, India) according to the manufacturer. The standard curve range was 5–2000 ng/L. The sensitivity was 2.36 ng/L. Intra-assay: CV < 9%; inter-assay: CV < 10%.

### 2.5. Statistical Analysis

The data collected in our study were analyzed using IBM SPSS 21 software. Because there is no study about serum ANGPTL8 levels in patients with AP in the literature, G-Power analysis was carried out using Cohen’s numbers. The smallest sample size was determined as 26 + 26, and total sample size as 52, with a margin of error of 0.05, an effect power of 80% and an effect size of 0.8. Normality control of continuous variables was evaluated with the Shapiro–Wilk test. Since the variables did not comply with normal distribution, non-parametric statistical methods were applied. Accordingly, the Mann–Whitney U test was used in the comparisons between two independent groups, and the Spearman Rho correlation coefficient was used to examine the relationship between two continuous variables. While determining the cut-off point for ANGPTL8, the receiver operating characteristic (ROC) curve analysis, area under the curve (AUC) and the DeLong test were used. *p* < 0.05 was considered statistically significant.

## 3. Results

The distribution of demographic and biochemical parameters and ANGPTL8 levels between groups is presented in Table 1. The present study comprised a total of 89 patients, of whom 39 were a healthy control group.

When we examined the relation of ANGPTL8 with the other biochemical parameters, we found that in the patient group, a negative, weakly significant relationship was observed between ANGPTL8 with glucose (r = −0.293; *p* = 0.039) and lipase (r = −0.285; *p* = 0.045). There was a statistically significant relation between the levels of ANGPTL8 and severe of AP (*p* < 0.05).

**Figure 1:**
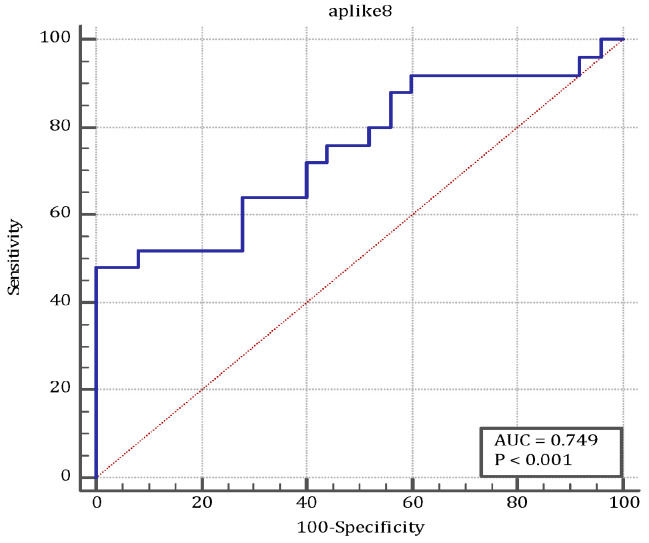
The ROC curve of ANGPTL8 levels according to the Glasgow–Imrie score.

**Figure 2:**
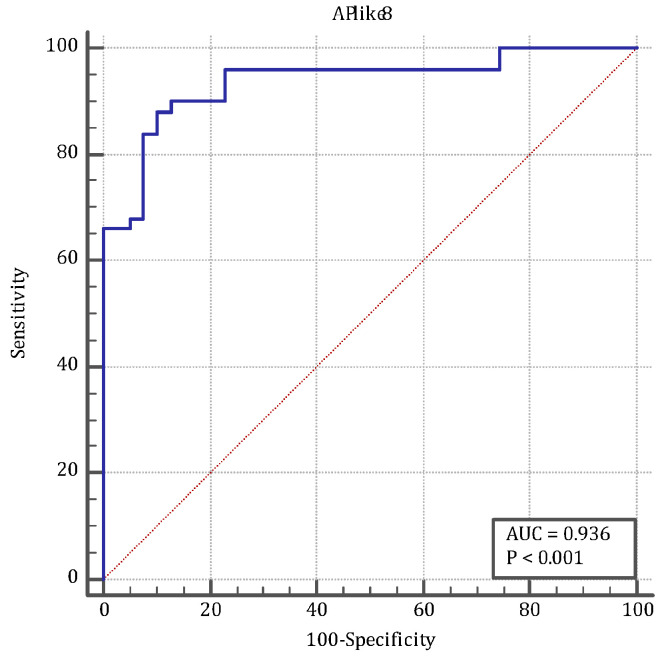
The ROC curve of ANGPTL8 levels to diagnose acute pancreatitis.

ANGPTL8 levels according to the Glasgow–Imrie score are presented in Table 2. Figure 1 shows the AUC according to the GI score, including 50 individuals. ANGPTL8 according to the GI score was calculated as 0.749 (95% CI: 0.606–0.861) (*p* < 0.001) (Table 3). The ROC curve of ANGPTL8 levels to diagnose AP is presented in Table 4 and Figure 2.

**Figure 3:**
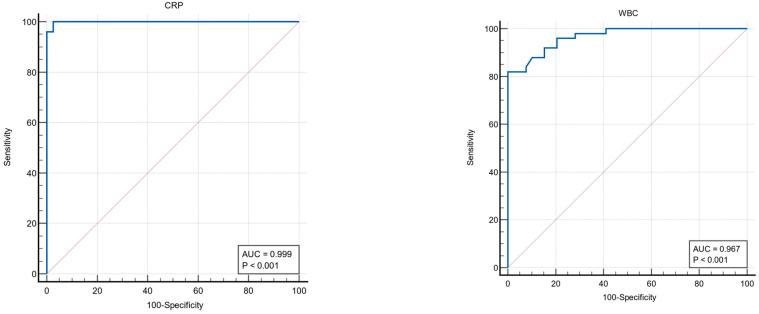
Diagnostic criteria of the ROC curve for CRP and WBC to diagnose acute pancreatitis.

ROC analysis results for important biochemical parameters showed that both parameters (CRP and WBC) had high discrimination power from patients with acute pancreatitis (Figure 3, Table 5).

Statistical significance for ANGPTL8 after adjustment for age and gender is presented in Table 6.

## 4. Discussion

The studies available in the literature have researched ANGPTL8 in many systems, but until now ANGPTL8 has not been studied in patients with AP. In the current study, levels of ANGPTL8 were markedly decreased in patients with AP. According to the GI score, ANGPTL8 levels were significantly higher in patients with a GI score < 3 than in patients with a GI score ≥ 3. According to the overall classification rate, ANGPTL8 showed the highest discriminatory power from healthy to AP with a sensitivity of 88.00% and specificity of 89.74%. This study showed that decreased ANGPTL8 levels were an independent predictor of disease severity in patients with AP. Novel therapeutic strategies targeting ANGPTL8, a modulator of LPL, could lead to progress in the treatment and prevention of AP. When ANGPTL8 levels are reduced, the uncontrolled inflammatory response may lead to tissue injury and worsen the severity of AP. ANGPTL8 could potentially influence the resolution of inflammation by modulating lipid metabolism, which in turn affects immune cell functions and inflammatory mediator production. ANGPTL8’s effects have been studied in various biological systems. This study applies existing knowledge to the specific context of AP, integrating the current literature into clinical practice and enhancing its applicability.

AP refers to sudden inflammation of the pancreas. Digestive enzymes are activated in the pancreatic interstitium and systemic circulation, resulting in autodigestion in pancreatic tissue, increased cytokine production, systemic inflammatory response syndrome (SIRS), and multiple organ failure [17]. Determining the severity of the disease is very important in terms of patient follow-up and predicting mortality.

Jung et al. [18] showed that ANGPTL4 expression was increased in the serum and pancreatic tissues; they also said that exogenous ANGPTL4 exacerbated pancreatic injury with elevated cytokine levels and apoptotic cell death. They also found that high ANGPTL4 enhanced macrophage activation and infiltration into the pancreas and suggested that targeting ANGPTL4 is a potential strategy for the treatment of AP. Catalano-Iniesta et al. [11] found a modest difference related to gender. These studies were the results expected when we considered the physiology of ANGPTL 4. When we considered the physiological mechanism of ANGPTL8, we expected ANGPTL8 levels to be high in AP. When we began our study, we expected to find similar results, but that was not the case. In our study, the level of ANGPTL8 was lower in AP than in the control group. We found that there was a negative statistically significant correlation between ANGPTL8 and severity of AP; this was exactly the opposite of our expectation. In this situation our hypothesis is as follows: it is likely to be due to the increased arachidonic acid level during pancreatitis inhibiting the expression of ANGPTL8, and, secondly, due to the low level of ANGPTL8, LPL activity is less affected; accordingly, it may be possible that increased free fatty acids following further hydrolysis of TG may predispose to pancreatitis. There was no significant difference between the control group and the AP group. This could suggest that TG levels in the blood might not be a primary differentiating factor between patients with AP and healthy controls, at least in the specific context of this study. However, several points could be important to consider. Although elevated TG levels are a well-known risk factor for AP, not all patients with AP present with elevated TG. Not all cases of AP are related to TG levels. Some cases might be triggered by factors like gallstones, alcohol, or medications, rather than hypertriglyceridemia. Patients having AP due to hypertriglyceridemia or alcohol consumption were not included in the study.

Another explanation for the decrease of ANGPTL8 in AP might be that the body’s compensatory mechanisms are at play. In AP, the liver, adipose tissue, and other organs could be altering their metabolic functions in ways that regulate TG levels, even if LPL or ANGPTL8 are dysregulated. So, despite potential disruptions in lipid metabolism, the overall TG level may not be considerably different. Also, it is known that ANGPTL8 acts together with ANGPTL3. There could be a mechanism through ANGPTL3 as well [19].

AP is an unpredictable disease early in its course. Healthcare providers need to acknowledge the difficulty in predicting the progression to severe disease for patients diagnosed with AP in the initial 24–48 h after admission. Despite extensive research efforts, severity scoring systems remain complex and generally require 48 h to provide accurate assessments. Moreover, when these systems can predict the severity, the patient’s condition is often already clearly severe, making the score less impactful in those instances. This is particularly evident with the Ranson, Modified Glasgow, Glasgow–Imrie, and APACHE II scoring systems. The American Gastroenterological Association (AGA) has prepared a guideline to assess the severity of AP [20,21,22]. Recommendations are as follows: (i) APACHE II can be used to define severity. (ii) Those with severe AP or comorbid disease should be hospitalized in the intensive care unit. (iii) Contrast-enhanced CT should be performed to evaluate pancreatic necrosis in patients with severe AP (APACHE II ≥ 8) or organ failure within the first 72 h. (iv) For patients who do not fulfill these criteria, the indication for CT should be clinically based. (v) Laboratory tests can be used to aid clinical judgment and APACHE II score. Of these tests, CRP > 15 mg/dL is preferred, especially in the first 48 h. By following these recommendations, clinician providers can better predict the severity of AP, facilitate early interventions, and improve patient outcomes.

We also identified that during AP, the cut-off level of ANGPTL8 according to the GI score helps us to predict those with a GI score < 3 100% correctly, but the rate of correctly predicting those with a GI score ≥ 3 was low and not significant. According to the overall classification rate, ANGPTL8 showed the highest discriminatory power from healthy to AP with a sensitivity of 88.00% and specificity of 89.74%. This research suggests that levels of ANGPTL8 could be an important biological marker for prognosis of AP. However, more clinical studies with many numbers of patients are needed to fully elucidate the relationship between ANGPTL8 and AP. ANGPTL8 plays a role in regulating lipid metabolism, although the exact mechanism through which it exerts its effects is still unclear. Further issues are yet to be clarified; for instance, whether ANGPTL8 serves as a lipoprotein that is embedded with lysosome/lipid-associated vesicles and secreted in plasma, and the physiological benefits and cell functions promoted by ANGPTL8 expression [23].

It has been reported that the data obtained from clinical studies so far have been inconsistent as to whether serum ANGPTL8 levels can be used as a biomarker for IR and related diseases [24,25,26,27,28,29,30,31]. Consistent with our findings, Gómez-Ambrosi et al. [18] reported that serum ANGPTL8 is decreased in human obesity, being further reduced in obesity-associated IR. ANGPTL8 levels are closely related to obesity-associated cardiometabolic risk factors, emerging as a potential biomarker of IR and T2DM. Although the involvement of ANGPTL8 in lipid regulation has been confirmed by several labs, its mechanism of action remains unclear [32,33,34,35,36]. Looking forward, further studies are needed to validate these findings in larger and more diverse patient populations. Future research should explore the mechanistic role of ANGPTL8 in AP pathophysiology and its potential as a therapeutic target. Additionally, longitudinal studies could help determine whether ANGPTL8 levels can serve as a reliable biomarker for disease progression or response to treatment in AP patients. The development of targeted therapies that modulate ANGPTL8 could offer new avenues for improving patient outcomes in AP.

Butyrylcholinesterase (BChE) is an alpha-glycoprotein found in various tissues, especially the liver. Decreased levels of BChE have been associated with higher mortality rates in liver transplant surgeries. Moreover, advanced cancer is often accompanied by mild to moderate inflammation and varying degrees of protein–energy malnutrition (PEM), which leads to a reduction in plasma BChE levels and an elevated risk of mortality. Verras and Mulita [37] investigated the relationship between BChE levels and the occurrence of surgical site infections (SSIs) following colorectal surgery in a prospective manner. The findings suggest that, even after accounting for known risk factors for SSI, lower and declining BChE levels on both the first and third day post-surgery are associated with a higher risk of developing SSI. Reduced BChE activity in blood plasma is associated with a shorter survival time in pancreatic cancer (PC) patients. BChE could serve as a useful tool for further stratifying patients into distinct prognostic risk categories. Decreased BChE activity in blood plasma may be associated with a shorter survival time in pancreatitis patients. Therefore, BChE could assist in determining the severity of pancreatitis [38].

In the current study, after adjusting for potential confounders such as age and gender, ANGPTL8 continued to show statistical significance in relation to AP. The statistical significance of ANGPTL8 after adjusting for age and gender strengthens the argument that it could be a valuable marker or factor in AP, highlighting its potential importance in clinical or research contexts. This indicates that the observed association between ANGPTL8 levels and AP remains robust and is not influenced by these demographic factors. The diagnostic performance of CRP and WBC values in distinguishing between patient and control groups was also examined. When the cut-off value for CRP was determined as >11, the test was found to have a very high discrimination power (AUC = 0.999; 95% CI: 0.957–1.00). At this value, the sensitivity was found to be 100% (92.9–100.0) and the specificity was found to be 97.4% (86.5–99.9), and the difference was statistically significant (*p* < 0.001). These results show that the CRP level showed a near-perfect performance in distinguishing between the patient and control groups. When the cut-off value for WBC was taken as >9900, the AUC value was calculated as 0.967 (0.905–0.993), and the test was found to have high accuracy. At this value, sensitivity was 82.0% (68.6–91.4) and specificity was 100% (91.0–100), and a statistically significant difference was observed. WBC also stands out as a very successful biomarker in distinguishing patients from healthy people, but its sensitivity is lower than CRP. Abu-Farha et al. [39] reported that ANGPTL8 showed a significant positive association with hsCRP, BMI, TG, LDL, HOMA-IR, and FBG. Age and ethnicity-adjusted ANGPTL8 levels increased with hsCRP in both MetS and non-MetS subjects, as well as across BMI groups. Their study showed that ANGPTL8 is increased in subjects with MetS, and it was significantly associated with hsCRP levels in different subgroups, highlighting its potential role in metabolic and inflammatory pathways. Both the current study and Abu-Farha et al. [39] highlight the significant role of ANGPTL8 in inflammation, but they focus on different clinical conditions. The current study emphasizes ANGPTL8’s potential as a valuable marker in AP, demonstrating its statistical significance even after adjusting for age and gender, which suggests its robustness in this context. In contrast, Abu-Farha et al. found ANGPTL8 to be positively associated with various metabolic markers, including hsCRP, BMI, and TG, particularly in subjects with MetS, emphasizing its role in metabolic and inflammatory pathways. While both studies suggest that ANGPTL8 could be an important biomarker, the current study focuses on its relevance to AP, whereas Abu-Farha et al. expand its significance to broader metabolic and inflammatory conditions. A recent study in 2025 also found a positive association between ANGPTL8 with inflammation, diabetes prevalence, and cardiovascular mortality [40].

### Strengths and Limitations of the Study

The effects of ANGPTL8 on AP have not been sufficiently explored yet, so this study fills a significant gap in the literature. It is an innovative study that will contribute to advancing the field. These strengths show that the study could make important contributions not only to scientific knowledge but also to clinical practices and treatment strategies for AP. The lack of research on ANGPTL8 in patients with AP presents a significant gap in the current literature, which may hold the potential for discovery of novel insights. One of the main advantages of investigating ANGPTL8 in this context is its potential role in regulating lipid metabolism, which is a critical factor in AP pathophysiology. Given ANGPTL8’s involvement in lipid homeostasis and its ability to influence metabolic pathways related to energy storage and inflammation, studying its role in AP could provide new perspectives on both the underlying mechanisms of the disease and potential therapeutic targets.

However, to fully understand these mechanisms, it is essential to assess control levels after the resolution of pancreatitis, which was a limitation of our study. Another limitation was the relatively small sample size, as it was a single-center study. Therefore, larger, multi-center studies are needed to draw more conclusive results.

## 5. Conclusions

ANGPTL8 might play a significant role in modulating the inflammatory response or metabolic dysfunction associated with AP. ANGPTL8 could act as a biomarker for identifying more severe cases of the disease, potentially helping to stratify patients based on their risk and guiding clinical decisions. The reduction in ANGPTL8 levels could indicate altered lipid metabolism, where LPL activity is deregulated, leading to an accumulation of free fatty acids and exacerbating pancreatic inflammation. This suggests that ANGPTL8 might have a protective role in regulating lipid homeostasis during AP. Its decrease could lead to worsened TG metabolism, contributing to the severity of the disease. ANGPTL8 remains a significant factor in AP even after adjusting for age and gender, highlighting its potential as a valuable marker in both clinical and research settings. Additionally, CRP and WBC are effective biomarkers in distinguishing between patients with AP and control groups, with CRP demonstrating near-perfect diagnostic performance. These findings emphasize the importance of these biomarkers in AP diagnosis. Since serum ANGPTL8 has not been previously studied in AP, our study is a first in the literature, and further research is needed to validate these findings.

## Figures and Tables

**Table 1:** Distribution of demographic and biochemical parameters and ANGPTL8 levels between groups.

	Control	Patient	
	Median [IQR]	Median [IQR]	*p*
Age (Years)	50 [43–64]	58.5 [49–66.3]	0.066
Gender (Male/Female) (%)	13/26 (33.3/66.7)	24/26 (48/52)	0.164 *
BMI (kg/m^2^)	26.2 [25.2–27.4]	26.2 [25.2–27.4]	0.429
CRP (mg/L)	3 [2–5]	88 [31.3–121.3]	**<0.001**
ALT (U/L)	19 [14–29]	59 [18.8–124.5]	**<0.001**
AST (U/L)	21 [17–26]	46.5 [21.5–209.5]	**<0.001**
Glucose (mg/dL)	87 [82–93]	131 [113–192]	**<0.001**
Urea (mg/dL)	22 [20–25]	38.5 [24–52.3]	**<0.001**
Creatinine (mg/dL)	0.7 [0.6–0.8]	0.9 [0.7–1.1]	**<0.001**
Albumin (g/L)	41 [40–43]	42.5 [38–45]	0.323
Calcium (mg/dL)	8.9 [8.7–9.1]	8.9 [8.2–9.3]	0.627
D.Bilirubin (mg/dL)	0.7 [0.4–0.8]	0.3 [0.1–1.1]	0.072
Triglyceride (mg/dL)	104 [97–132]	100 [80.5–130.5]	0.331
LDL (mg/dL)	99 [89–109]	100 [88.5–111]	0.882
Leukocyte (10^3^ µ/L)	5500 [4700–7300]	13,800 [11,150–16,825]	**<0.001**
Hemoglobin (g/L)	13.4 [12.3–14.2]	14 [12.8–15.1]	0.129
Platelet (10^3^ µ/L)	318,000 [246,000–376,000]	235,000 [194,500–313,250]	**0.011**
ANGPTL8 (ng/L)	285.9 [202–540]	110 [74–163]	**<0.001**

*p*: Mann–Whitney U test; * Chi-squared test.

**Table 2:** ANGPTL8 levels according to the Glasgow–Imrie score.

ANGPTL8	Mean ± SD	Median [IQR]	Min–Max	*p*
GI score < 3	149.05 ± 80.5	127.7 [100.2–174.4]	75.52–490	0.003
GI score ≥ 3	104.49 ± 89.74	86.1 [48.93–127.75]	21.51–472	

*p*: Mann Whitney U test.

**Table 3:** Cut-off level of ANGPTL8 according to the Glasgow–Imrie score.

	Cut-Off	AUC (95% CI)	Sensitivity (95% CI)	Specificity (95% CI)	*p*
ANGPTL8	≤70.9	0.749 (0.606–0.861)	48.0 (27.8–68.7)	100.0 (86.3–100.0)	<0.001

**Table 4:** Cut-off level for ANGPTL8 according to the overall classification rate.

	Cut-Off	AUC (95% CI)	Sensitivity (95% CI)	Specificity (95% CI)	*p*
ANGPTL8	≤179.2	0.936 (0.864–0.977)	88.00 (75.7–95.5)	89.74 (75.8–97.1)	<0.001

**Table 5:** Cut-off values, sensitivity, and specificity values by ROC analysis for CRP and WBC values indicating acute pancreatitis.

	Cut-Off	AUC (95% CI)	Sensitivity (95% CI)	Specificity (95% CI)	*p*
CRP	>11	0.999 (0.957–1.00)	100 (92.9–100.0)	97.4 (86.5–99.9)	<0.001
WBC	>9900	0.967 (0.905–0.993)	82.0 (68.6–91.4)	100 (91.0–100)	<0.001

**Table 6:** Statistical significance of ANGPTL8 after adjusting for age and gender.

	Control			Patient				
	Mean ± SD	Median [IQR]	Min–Max	Mean ± SD	Median [IQR]	*p*	*p*	p_adj_
ANGPTL8	476.46 ± 461.48	285.9 [201.9–539.6]	139.4–2400	126.77 ± 87.32	109.45 [74.37–162.95]	21.51–490.2	**<0.001**	**<0.001**

*p*: Mann–Whitney U test, p_adj_: covariance analysis.

## Data Availability

The data underlying this article are available in the article. If needed, please contact the corresponding author. The email address is perihangumuskaya@hotmail.com.

## References

[1] Wang Y., Chen H., Li H., Zhang J., Gao Y. (2013). Effect of angiopoietin-like protein 4 on rat pulmonary microvascular endothelial cells exposed to LPS. Int. J. Mol. Med..

[2] Banks P.A., Bollen T.L., Dervenis C., Gooszen H.G., Johnson C.D., Sarr M.G., Tsiotos G.G., Vege S.S. (2013). Classification of acute pancreatitis—2012: Revision of the Atlanta classification and definitions by international consensus. Gut.

[3] Chung H.S., Lee M.J., Hwang S.Y., Lee H.J., Yoo H.J., Seo J.A., Kim S.G., Kim N.H., Baik S.H., Choi D.S. (2016). Circulating angiopoietin-like protein 8 (ANGPTL8) and ANGPTL3 concentrations in relation to anthropometric and metabolic profiles in Korean children: A prospective cohort study. Cardiovasc. Diabetol..

[4] Guo C., Zhao Z., Deng X., Chen Z., Tu Z., Yuan G. (2019). Regulation of angiopoietin-like protein 8 expression under different nutritional and metabolic status. Endocr. J..

[5] Guo C., Wang C., Deng X., He J., Yang L., Yuan G. (2021). ANGPTL8 in metabolic homeostasis: More friend than foe?. Open Biol..

[6] Jiao X., Yang Y., Li L., Yu H., Yang Y., Li J., Du Y., Zhang J., Hu C., Qin Y. (2020). Angiopoietin-like protein 8 accelerates atherosclerosis in ApoE^−/−^ mice. Atherosclerosis.

[7] Chen Y.Q., Pottanat T.G., Siegel R.W., Ehsani M., Qian Y.W., Zhen E.Y., Regmi A., Roell W.C., Guo H., Luo M.J. (2020). Angiopoietin-like protein 8 differentially regulates ANGPTL3 and ANGPTL4 during postprandial partitioning of fatty acids. J. Lipid Res..

[8] Ebert T., Kralisch S., Hoffmann A., Bachmann A., Lössner U., Kratzsch J., Blüher M., Stumvoll M., Tönjes A., Fasshauer M. (2014). Circulating angiopoietin-like protein 8 is independently associated with fasting plasma glucose and type 2 diabetes mellitus. J. Clin. Endocrinol. Metab..

[9] Yi P., Park J.S., Melton D.A. (2013). Betatrophin: A hormone that controls pancreatic β cell proliferation. Cell.

[10] Zhang L., Shannon C.E., Bakewell T.M., Abdul-Ghani M.A., Fourcaudot M., Norton L. (2020). Regulation of ANGPTL8 in liver and adipose tissue by nutritional and hormonal signals and its effect on glucose homeostasis in mice. Am. J. Physiol. Endocrinol. Metab..

[11] Catalano-Iniesta L., Robledo V.S., Iglesias-Osma M.C., Albiñana A.G., Carrero S., Blanco E.J., Carretero-Hernández M., Carretero J., García-Barrado M.J. (2020). Evidences for Expression and Location of ANGPTL8 in Human Adipose Tissue. J. Clin. Med..

[12] Mısırlıoglu N.F., Ergun S., Kucuk S.H., Himmetoglu S., Ozen G.D., Sayili U., Uzun N., Uzun H. (2025). The Importance of Resolvin D1, LXA4, and LTB4 in Patients with Acute Pancreatitis Due to Gallstones. Medicina.

[13] Pekmezci Y., Ergun S., Turgut B.C., Dumur S., Sayili U., Uzun H., Pekmezci S., Velidedeoglu M. (2025). The Role of Resolvin D1 in the Differential Diagnosis of Pancreatic Ductal Adenocarcinoma and Acute Pancreatitis: A Case-Control Study. Medicina.

[14] Mititelu A., Grama A., Colceriu M.C., Benţa G., Popoviciu M.S., Pop T.L. (2024). Role of Interleukin 6 in Acute Pancreatitis: A Possible Marker for Disease Prognosis. Int. J. Mol. Sci..

[15] Efgan M.G., Karakaya Z., Kanter E., Kırık S., Tekindal M.A. (2024). Can CONUT and PNI Scores Predict Necrotizing Pancreatitis in Acute Pancreatitis Patients Presenting to the Emergency Department?. J. Clin. Med..

[16] Buxbaum J., Quezada M., Chong B., Gupta N., Yu C.Y., Lane C., Da B., Leung K., Shulman I., Pandol S. (2018). The Pancreatitis Activity Scoring System predicts clinical outcomes in acute pancreatitis: Findings from a prospective cohort study. Am. J. Gastroenterol..

[17] Feng P., He C., Liao G., Chen Y. (2017). Early enteral nutrition versus delayed enteral nutrition in acute pancreatitis: A PRISMA compliant systematic review and meta-analysis. Medicine.

[18] Jung K.H., Son M.K., Yan H.H., Fang Z., Kim J., Kim S.J., Park J.H., Lee J.E., Yoon Y.C., Seo M.S. (2020). ANGPTL4 exacerbates pancreatitis by augmenting acinar cell injury through upregulation of C5a. EMBO Mol. Med..

[19] Rafaqat S., Radoman-Vujačić I., Patoulias D., Khurshid H., Klisić A. (2024). Adipokines and their role in acute pancreatitis. J. Med. Biochem..

[20] Forsmark C.E., Baillie J., AGA Institute Clinical Practice and Economics Committee, AGA Institute Governing Board (2007). AGA Institute technical review on acute pancreatitis. Gastroenterology.

[21] Tenner S., Vege S.S., Sheth S.G., Sauer B., Yang A., Conwell D.L., Yadlapati R.H., Gardner T.B. (2024). American College of Gastroenterology Guidelines: Management of Acute Pancreatitis. Am. J. Gastroenterol..

[22] Chauhan R., Saxena N., Kapur N., Kardam D. (2022). Comparison of modified Glasgow-IMRIE, Ranson, and Apache II scoring systems in predicting the severity of acute pancreatitis. Pol. J. Surg..

[23] Tseng Y.H., Yeh Y.H., Chen W.J., Lin K.H. (2014). Emerging regulation and function of betatrophin. Int. J. Mol. Sci..

[24] Gómez-Ambrosi J., Pascual E., Catalán V., Rodríguez A., Ramírez B., Silva C., Gil M.J., Salvador J., Frühbeck G. (2014). Circulating betatrophin concentrations are decreased in human obesity and type 2 diabetes. J. Clin. Endocrinol. Metab..

[25] Maurer L., Brachs S., Decker A.-M., Brachs M., Leupelt V., von Schwartzenberg R.J., Ernert A., Bobbert T., Krude H., Spranger J. (2016). Weight Loss Partially Restores Glucose-Driven Betatrophin Response in Humans. J. Clin. Endocrinol. Metab..

[26] Gokulakrishnan K., Manokaran K., Pandey G.K., Amutha A., Ranjani H., Anjana R.M., Mohan V. (2015). Relationship of betatrophin with youth onset type 2 diabetes among Asian Indians. Diabetes Res. Clin. Pract..

[27] Tuhan H., Abacı A., Anık A., Çatlı G., Küme T., Çalan Ö.G., Acar S., Böber E. (2016). Circulating betatrophin concentration is negatively correlated with insulin resistance in obese children and adolescents. Diabetes Res. Clin. Pract..

[28] Chen X., Lu P., He W., Zhang J., Liu L., Yang Y., Liu Z., Xie J., Shao S., Du T. (2015). Circulating betatrophin levels are increased in patients with type 2 diabetes and associated with insulin resistance. J. Clin. Endocrinol. Metab..

[29] Fenzl A., Itariu B.K., Kosi L., Fritzer-Szekeres M., Kautzky-Willer A., Stulnig T.M., Kiefer F.W. (2014). Circulating betatrophin correlates with atherogenic lipid profiles but not with glucose and insulin levels in insulin-resistant individuals. Diabetologia.

[30] Hu H., Sun W., Yu S., Hong X., Qian W., Tang B., Wang D., Yang L., Wang J., Mao C. (2014). Increased circulating levels of betatrophin in newly diagnosed type 2 diabetic patients. Diabetes Care..

[31] Wang S., Hong X., Tu Z., Yuan G. (2017). Angiopoietin-like protein 8: An attractive biomarker for the evaluation of subjects with insulin resistance and related disorders. Diabetes Res. Clin. Pract..

[32] Zhang Y., Li S., Donelan W., Xie C., Wang H., Wu Q., Purich D.L., Reeves W.H., Tang D., Yang L.J. (2016). Angiopoietin-like protein 8 (betatrophin) is a stress-response protein that down-regulates expression of adipocyte triglyceride lipase. Biochim. Biophys. Acta.

[33] Ren G., Kim J.Y., Smas C.M. (2012). Identification of RIFL, a novel adipocyte-enriched insulin target gene with a role in lipid metabolism. Am. J. Physiol. Endocrinol. Metab..

[34] Zhang R. (2012). Lipasin, a novel about nutritionally regulated liver-enriched factor that regulates serum triglyceride levels. Biochem. Biophys. Res. Commun..

[35] Quagliarini F., Wang Y., Kozlitina J., Grishin N.V., Hyde R., Boerwinkle E., Valenzuela D.M., Murphy A.J., Cohen J.C., Hobbs H.H. (2012). Atypical angiopoietin-like protein that regulates ANGPTL3. Proc. Natl. Acad. Sci. USA.

[36] Wang Y., Quagliarini F., Gusarova V., Gromada J., Valenzuela D.M., Cohen J.C., Hobbs H.H. (2013). Mice lacking ANGPTL8 (Betatrophin) manifest disrupted triglyceride metabolism without impaired glucose homeostasis. Proc. Natl. Acad. Sci. USA.

[37] Verras G.I., Mulita F. (2024). Butyrylcholinesterase levels correlate with surgical site infection risk and severity after colorectal surgery: A prospective single-center study. Front. Surg..

[38] Klocker E.V., Barth D.A., Riedl J.M., Prinz F., Szkandera J., Schlick K., Kornprat P., Lackner K., Lindenmann J., Stöger H. (2020). Decreased Activity of Circulating Butyrylcholinesterase in Blood Is an Independent Prognostic Marker in Pancreatic Cancer Patients. Cancers.

[39] Abu-Farha M., Abubaker J., Al-Khairi I., Cherian P., Noronha F., Kavalakatt S., Khadir A., Behbehani K., Alarouj M., Bennakhi A. (2016). Circulating angiopoietin-like protein 8 (betatrophin) association with HsCRP and metabolic syndrome. Cardiovasc. Diabetol..

[40] Silbernagel G., Chen Y.Q., Li H., Lemen D., Wen Y., Zhen E.Y., Rief M., Kleber M.E., Delgado G.E., Sarzynski M.A. (2025). Associations of Circulating ANGPTL3, C-Terminal Domain-Containing ANGPTL4, and ANGPTL3/8 and ANGPTL4/8 Complexes with LPL Activity, Diabetes, Inflammation, and Cardiovascular Mortality. Circulation.

